# Plasma-Induced Catalyst Support Defects for the Photothermal Methanation of Carbon Dioxide

**DOI:** 10.3390/ma14154195

**Published:** 2021-07-28

**Authors:** Salina Jantarang, Simone Ligori, Jonathan Horlyck, Emma C. Lovell, Tze Hao Tan, Bingqiao Xie, Rose Amal, Jason Scott

**Affiliations:** Particles and Catalysis Research Group, School of Chemical Engineering, The University of New South Wales, Sydney, NSW 2052, Australia; salina.j@chula.ac.th (S.J.); simligori@gmail.com (S.L.); j.horlyck@outlook.com (J.H.); tze_hao.tan@unsw.edu.au (T.H.T.); bingqiao.xie@unsw.edu.au (B.X.); r.amal@unsw.edu.au (R.A.)

**Keywords:** photothermal carbon dioxide methanation, plasma treatment, helium plasma, nickel catalyst, titania, defects

## Abstract

The presence of defects in a catalyst support is known to benefit catalytic activity. In this work, a He-plasma treatment-based strategy for introducing and stabilising defects on a Ni/TiO_2_ catalyst for photothermal CO_2_ hydrogenation was established. The impact of pretreatment step sequence—which comprised He-plasma treatment and reduction/passivation—on defect generation and stabilisation within the support was evaluated. Characterisation of the Ni/TiO_2_ catalysts indicated that defects created in the TiO_2_ support during the initial plasma treatment stage were then stabilised by the reduction/passivation process, (P-R)Ni/TiO_2_. Conversely, performing reduction/passivation first, (R-P)Ni/TiO_2_, invoked a resistance to subsequent defect formation upon plasma treatment and consequently, poorer photothermal catalytic activity. The plasma treatment altered the metal-support interaction and ease of catalyst reduction. Under photothermal conditions, (P-R)Ni/TiO_2_ reached the highest methane production in 75 min, while (R-P)Ni/TiO_2_ required 165 min. Decoupling the impacts of light and heat indicated thermal dominance of the reaction with CO_2_ conversion observed from 200 °C onwards. Methane was the primary product with carbon monoxide detected at 350 °C (~2%) and 400 °C (~5%). Overall, the findings demonstrate the importance of pretreatment step sequence when utilising plasma treatment to generate active defect sites in a catalyst support.

## 1. Introduction

The transformation of carbon dioxide (CO_2_) to valuable hydrocarbon products is a promising approach to mitigate CO_2_ emissions to the atmosphere [1,2]. Amongst the fuels and chemicals which can be produced, methane is a desirable product as it can be used directly within our existing natural gas infrastructure [3,4]. One approach to driving the methanation reaction is through exploiting photothermal conditions, which relies on a temperature increase from light absorption by the catalyst. The photothermal conditions allow the reaction to occur without external heating and promote a sustainable approach for catalytic activity.

To date, studies surrounding photothermal CO_2_ methanation have focused on understanding the role of light and catalyst design, encompassing different active metals and catalysts [5,6,7]. Amongst the active metals studied for thermal CO_2_ hydrogenation, nickel (Ni) has been probed expansively due to its performance in CO_2_ hydrogenation and relative abundance [3,8,9,10,11]. With regard to catalyst supports, titania (TiO_2_) has been studied comprehensively for thermal CO_2_ methanation [9,12,13]. TiO_2_, a semiconductor support, can harness light primarily in the ultraviolet (UV) region [14]. While catalysts have been prepared with varied metals and supports, the methods of catalyst modification, such as to induce defects, have not been reported extensively. The photothermal reaction is influenced by factors such as light harnessing, ease of catalyst reduction, and interaction with CO_2_ and H_2_ [15,16,17]. Therefore, pretreatments to alter catalyst structure can enhance the reaction.

One approach to modifying catalytic properties is by pretreating the catalyst [18,19,20,21]. Plasma treatment is capable of rapidly form defects without requiring additional chemicals or complex reactor set-ups. Non-thermal plasma pretreatment induce defects by bombarding the catalyst surface with excited species, typically easily ionised gases such as argon (Ar) or helium (He) [22,23]. This surface bombardment can result in a range of physical and chemical changes to the catalyst surface including etching, reducing the surface and altering metal deposit size and support interaction [22,23]. Plasma pretreatment has shown significant impacts on both metal oxide supports alone, as well as metal-supported catalysts. For example, Horlyck et al. examined the use of He plasma pretreatment on TiO_2_/SiO_2_ composites and found a significant increase in defect formation as a result of prolonged plasma treatment [24]. In the case of metal-supported catalysts, the effect of cold Ar plasma treatment on Pt/CeO_2_ for the water–gas shift reaction has been studied. Compared to calcined Pt/CeO_2_, the plasma treatment step altered the Pt species and resulted in an increase in electron density, stronger Pt-Ce interaction, and higher CO adsorption [25].

Plasma treatment, as a means of defecting catalysts and altering Ni properties, has not been investigated for pretreating catalysts used in the photothermal methanation of CO_2_. Overall, applying a plasma at different stages of catalyst preparation has been reported to impact material characteristics such as structural properties and metal-support interaction; subsequently influencing the catalytic performance.

In this work, plasma treatment was utilised to induce defects on Ni/TiO_2_ catalysts for the photothermal conversion of CO_2_ to methane. A TiO_2_ support was prepared by flame spray pyrolysis (FSP) and loaded with Ni. Plasma treatment was applied to either: (i) as-prepared NiO/TiO_2_ prior to reduction/passivation; or (ii) after reduction/passivation of the as-prepared NiO/TiO_2_. The plasma pretreatment can induce defects in the TiO_2_ support as well as influence the Ni catalyst properties. Its application at different points within the catalyst preparation process enables us to examine its influence on the Ni/TiO_2_ catalyst characteristics and their subsequent catalyst activity. The Ni/TiO_2_ catalysts were assessed for CO_2_ reduction under photothermal conditions in a batch-circulated reactor system, where light was the sole driving force for the reaction. To decouple the influence of light and heat on the catalytic process, the reaction was undertaken in a continuous flow reactor system. Within the continuous flow reactor, catalyst activity/selectivity under only either thermal stimulus or combined thermal-light stimuli, can be determined. The materials were characterised to gain an appreciation of the impact plasma treatment had on inducing defects in the TiO_2_ support and altering the Ni catalyst properties, and the ensuing catalytic activity for photothermal CO_2_ methanation.

## 2. Materials and Methods

### 2.1. Catalyst Synthesis

TiO_2_ was prepared by flame spray pyrolysis (FSP), with the synthesis conditions described elsewhere [26]. Briefly, titanium (IV) isopropoxide (97%, Sigma-Aldrich, St. Louis, MO, USA) was mixed with absolute ethanol (Chem-Supply, Adelaide, Australia) to form a 1.26 M precursor solution. The mixture was then fed into the flame via a syringe pump at a rate of 5 mL/min. A 5 L/min O_2_ sheath (oxygen, >99.9%, Coregas, Yennora, Australia) aided upward movement of the synthesised particles into the vacuum hood. The flame was supported with a mix of 1.5 L/min CH_4_ (>99.95%, Coregas) and 3.2 L/min O_2_. The precursor was dispersed using a 5 L/min gas flow of O_2_, with the pressure drop across the nozzle maintained at 150 kPa. A vacuum pump connected to a Whatmann filter paper located above the flame was used to collect the product.

NiO/TiO_2_ catalyst was prepared via impregnation and calcination. During the synthesis, nickel (II) nitrate hexahydrate (99.999%, Sigma-Aldrich) was dissolved in Milli-Q water (18.2 MΩ·cm, Merck Millipore, Billerica, MA, USA) to form a 0.3 M solution. The FSP-prepared TiO_2_ was added to the Ni solution and stirred at 120 °C until a paste formed. The paste was further dried at 110 °C in an oven for 16 h and then ground with a mortar and pestle. The powder was loaded into a glass reactor with a 10 mm internal diameter and calcined at 400 °C for 3 h under dry air (Coregas, 50 mL/min) at ramp rate of 5 °C/min.

The NiO/TiO_2_ catalysts were reduced and passivated in a Micromeritics Autochem 2910 (Micromeritics, Norcross, GA, USA). For the reduction step, ~300 mg of NiO catalyst was reduced in 10.18% H_2_/Ar (20 mL/min) ramped to 500 °C at 5 °C/min, held for 1 h, and then cooled under Ar. The reduced Ni catalyst was then passivated under 10 mL/min 0.97% O_2_/He (Coregas) for 12 h at room temperature, after which it was ground with a mortar and pestle.

Plasma treatment was undertaken using a Dielectric Barrier Discharge (DBD) plasma system (CTP-2000K Plasma Generator, Corona Lab, Nanjing, China), with a discharge gap of ~8 mm, as reported elsewhere [24]. Typically, ~250 mg of catalyst was loaded into the plasma chamber and subjected to plasma under a 30 mL/min He (>99.996%, Coregas) flow for 20 min. Plasma treatment was conducted on either: (i) the as-prepared NiO/TiO_2_ prior to reduction and passivation; or (ii) samples that had been initially reduced and passivated. The catalysts are labelled based on the pretreatment order where reduced/passivated and then plasma treated is (R-P)Ni/TiO_2_, plasma treated and then reduced/passivated is (P-R)Ni/TiO_2_. A catalyst that has been only reduced/passivated (i.e., no plasma treatment) is referred to as (R)Ni/TiO_2_. A catalyst that has been only plasma-treated (i.e., no reduction/passivation) is referred to as (P)NiO/TiO_2_.

### 2.2. Catalyst Characterisation

To determine the actual Ni loading on the catalyst, the as-prepared NiO/TiO_2_ was digested in aqua regia using microwave assistance and subject to inductively coupled plasma optical emission spectrometry (ICP–OES) using a PerkinElmer OPTIMA 7300 ICP-OES instrument (PerkinElmer, Waltham, MA, USA).

Specific surface area (SSA) was measured using N_2_ adsorption/desorption isotherms (Micromeritics Tristar 3030) at −196 °C. The samples were pretreated under vacuum for 3 h at 150 °C prior to analysis. The SSA was determined by the Brunauer–Emmet–Teller (BET) method. Material phases and crystallinity were analysed using X-ray diffraction (XRD) with a PANalytical Xpert Multipurpose X-ray Diffraction System (MPD) (Malvern, UK). The XRD instrument was set at 45 kV and 40 mA with a Cu Kα source. The pattern was collected over the range of 2θ = 20°–100° with a scan rate of 0.01°/min and a step size of 0.026°. The Scherrer equation was used to calculate the crystal size using the 100% intensity peak and a shape factor of 0.9.

As light absorbance by the Ni catalysts is critical for driving the photothermal methanation reaction, the materials were characterised by ultraviolet-visible-near infrared (UV-vis-NIR) spectrometry using a Shimadzu UV-Vis 3600 (Shimadzu, Kyoto, Japan). The collected reflectance spectra were converted to absorbance spectra via the Kubelka–Munk equation, referenced to barium sulphate. Subsequently, the converted spectra of nickel-loaded samples were normalised against absorbance of TiO_2_.

Catalyst reducibility was characterised using a Micromeritics Autochem 2910 (Micromeritics, Norcross, GA, USA). For the hydrogen-temperature programmed reduction (H_2_–TPR) procedure, approximately 50 mg of catalyst was loaded into a quartz U-tube, supported by a plug of quartz wool. The catalyst was pretreated under an argon (>99.997%, Coregas) flow (20 mL/min) where it was heated to 150 °C at a rate of 10 °C/min and held for 0.5 h. The sample was then cooled to 50 °C prior to introducing 10.18% H_2_/Ar (Coregas) at 20 mL/min. Heating from 50 °C to 700 °C at a ramp rate of 5 °C/min was applied and the H_2_ consumed was measured by a TCD. CO_2_-temperature programmed desorption (CO_2_–TPD) experiments were also conducted on the Micromeritics Autochem 2910. Here, approximately 50 mg of sample was placed into the quartz U-tube. The catalyst was initially reduced in 10.18% H_2_/Ar (20 mL/min) ramped at 5 °C/min up to 500 °C, held for 1 h, and cooled to 50 °C in 20 mL/min He. The sample was next exposed to 20 mL/min CO_2_ (>99.5%, Coregas) for 1 h at 50 °C after the gas flow was swapped to He for 1 h. The CO_2_–TPD was conducted in 20 mL/min He from 50 °C to 700 °C at a ramp rate 10 °C/min.

Surface species present on the pretreated Ni/TiO_2_ catalysts were identified by X-ray photoelectron spectroscopy (XPS). Shifts in binding in energy were referenced to the Carbon 1s peak (284.8 eV) and the spectra were collected using an ESCALAB 250Xi (Thermo Scientific, Waltham, MA, USA) equipped with an Al Kα X-ray source. Transmission electron microscopy (TEM) images and energy-dispersive spectroscopy (EDS) mapping were taken by a JEOL JEM-ARM200F microscope (JEOL Ltd., Tokyo, Japan), operating at 200 kV. Prior to the imaging, the Ni/TiO_2_ catalysts were ultrasonically dispersed in ethanol and drop-cast onto lacy carbon-coated copper grids. Defects present in the TiO_2_ were evaluated using electron paramagnetic resonance (EPR) and Raman spectroscopies. EPR was conducted at −153 °C (cooled by liquid nitrogen) using 20 mg of sample loaded in a 4 mm (internal diameter) quartz tube and analysed by a Bruker EMX X-Band ESR spectrometer (Bruker, Billerica, MA, USA). Raman spectroscopy was performed using a Renishaw inVia 2 Raman Microscope (532 nm) (Renishaw, Wotton-under-Edge, UK).

### 2.3. Activity Tests

Two styles of activity testing were conducted to gain an understanding of the impacts of plasma treatment on catalyst performance for the photothermal methanation of CO_2_: (i) the first utilised a batch-circulated reactor system; (ii) the second employed a continuous flow reactor system where the effects of light and heat on catalyst performance could be decoupled. Details on the two reactor configurations and associated catalyst activity/selectivity evaluation are available in an earlier study [16].

In the case of the batch-circulated reactor system, illumination was provided from the top of the reactor using a 300 W Xenon lamp (Peccell CX-04E with an Eagle R300-3J lamp housing) (Peccell Technologies, Yokohama, Japan). No other heat source was employed. The catalyst was supported on a glass fibre filter (Merck Millipore, Billerica, MA, USA) within the reactor. To load the catalyst, 100 mg of sample was ultrasonically dispersed in Milli-Q water and drop-cast on to a 7 cm^2^ region of the filter, dried and then placed in the reactor. A reactant mixture comprising ~15 kPa CO_2_ and ~60 kPa hydrogen (H_2_, >99.99%, Coregas) was injected into, and continuously circulated around, the reactor. The reactor was illuminated using the Xenon lamp (20 A current output) to initiate the photothermal methanation reaction. Reactants and products were analysed using a gas chromatograph (Shimadzu GC-2010 Plus with a Supelco Carboxen 1010 column) (Shimadzu, Kyoto, Japan). The gas chromatograph was equipped with a methaniser, flame ionisation detector (FID) and TCD. The change in temperature of the catalyst was monitored using a thermocouple in direct contact with the catalyst-loaded filter.

In the case of the continuous flow reactor system, heat was supplied from beneath the reactor using a tube furnace. The catalyst was illuminated by a Xenon lamp (300 W, 20 A current output) through a quartz window in the top of the reactor. The catalyst (100 mg) was loaded on an (1.5 cm × 1.5 cm) aluminium plate and placed in the reactor facing upwards. Under thermal-only reaction conditions, the temperature increased from 50–400 °C, at 50 °C intervals, under a 4 mL/min CO_2_ and 16 mL/min H_2_ reactant flow. The temperature was ramped at 2.5 °C/min and held for 1 h at each temperature interval. The impact of light was studied by replicating the thermal reaction conditions with the addition of catalyst illumination from above. Reactants and products from the reactor effluent were identified using a Shimadzu GC-2010 gas chromatograph (Shimadzu, Kyoto, Japan) equipped with an Agilent J&W HP-PLOT Q capillary column, methaniser and FID. A schematic of the apparatus can be found in Tan et al. [27].

## 3. Results and Discussion

### 3.1. Catalyst Properties 

The structural properties of Ni/TiO_2_ are displayed in Table 1. As determined from ICP-OES, the actual Ni loading was 9.2 wt.%, close to the nominal 10 wt.% loading. The as-prepared FSP TiO_2_ had a SSA of 104 m^2^/g. The type III N_2_ adsorption/desorption isotherms of TiO_2_ (Figure S2) indicates a non-porous structure. The pore size distribution (Figure S2) shows a maximum at approximately 3.46 nm for TiO_2_. After loading the TiO_2_ with Ni and subsequent calcination (NiO/TiO_2_), the SSA decreased to 66 m^2^/g. Plasma-treating the NiO/TiO_2_ ((P)NiO/TiO_2_) saw the SSA increase slightly to 75 m^2^/g. Both NiO/TiO_2_ and (P)NiO/TiO_2_ displayed type V isotherms. Two peaks, centred at 4.2–5.1 nm and 8.7–9.1 nm, were observed in the pore size distribution of the nickel catalysts. As the catalysts are non-porous, the porosity observed from N_2_ adsorption/desorption isotherm is likely to stem from spaces between the particles. The catalyst structure and metal distribution are discussed at the end of this section.

The crystal phase and size of the TiO_2_ support and Ni deposits were determined from the XRD patterns (Figure S2). Both anatase and rutile TiO_2_ were detected; however, the anatase phase was dominant, which is consistent with TiO_2_ synthesised by FSP [26]. The TiO_2_ crystal phase was not affected by plasma treatment or Ni addition and calcination. Plasma treatment had a minimal impact on the NiO crystallite size (NiO/TiO_2_: 7.7 nm, (P)NiO/TiO_2_: 9.3 nm) and the TiO_2_ crystal size (21 nm) remained unchanged. Upon reduction/passivation, the Ni crystal sizes were similar ((P-R)Ni/TiO_2_: 13.7 nm, (R)Ni/TiO_2_: 13.1 nm, (R-P)Ni/TiO_2_: 12.7 nm), indicating that the plasma treatment had little impact on this characteristic. The BET and XRD results indicate the plasma pretreatment does not impart any undesirable morphological changes.

**Figure 1:**
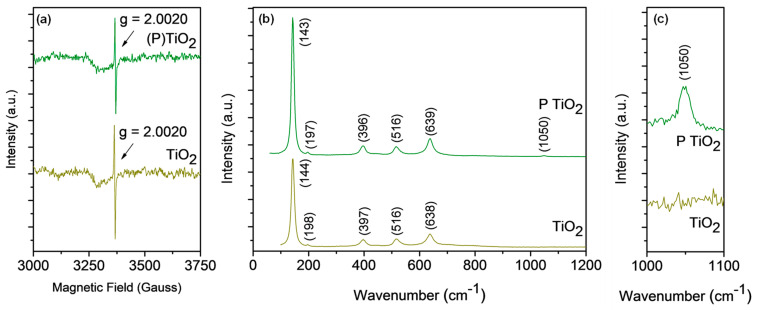
(**a**) EPR spectra of TiO_2_ and plasma-treated TiO_2_ ((P)TiO_2_); Raman spectra of TiO_2_ and (P)TiO_2_ (**b**) 0−1200 cm^−1^ and (**c**) magnified spectra at 1000−1100 cm^−1^. P = plasma treated.

As plasma treatment has the potential to induce defects on the TiO_2_ support, the as-prepared, plasma-treated and reduced/passivated TiO_2_ (without Ni loading) were studied by EPR and Raman spectroscopy (Figure 1). The EPR spectrum exhibits a peak at g = 2.0020 for the as-prepared TiO_2_, associated with the presence of oxygen vacancies [28,29]. The peak representing Ti^3+^ (g ≈ 1.94) was not observed [28]. The EPR spectrum remains unchanged after plasma treating the TiO_2_ ((P)TiO_2_), with no shift in the g = 2.0020 peak position and no new peaks appearing. Similarly, the EPR spectrum for the passivated/reduced sample (Figure S3a) remains unchanged.

Raman spectroscopy was used to further understand the influence of plasma treatment on the TiO_2_ (Figure 1b,c). Peaks corresponding to the anatase phase of TiO_2_ were evident for both samples (143/144 cm^−1^, 396/397 cm^−1^, 516 cm^−1^, and 638/639 cm^−1^) [30,31]. This is consistent with the presence of anatase from XRD (Figure S2). The peak at 143/144 cm^−1^ is the linear combination of asymmetric bending of O–Ti–O bonds, while 197/198 cm^−1^ can be attributed to the presence of the brookite phase [30]. Consistent with the XRD results, no significant peak shifts or broadening was evident as a consequence of the plasma treatment, indicating no major structural changes. A peak at 1050 cm^−1^ appeared for (P)TiO_2_, which is not commonly observed in TiO_2_. Surmacki et al. [32] observed a peak at 1048 cm^−1^ in an electron beam irradiated N-doped TiO_2_ sample which was not observed in the non-irradiated sample. They attributed it to a new species from the interaction of the TiO_2_ and N-dopant under irradiation, although it was not assigned to a specific feature of TiO_2_. Plasma treatment of FSP SiO_2_–TiO_2_ saw the emergence of a new Raman peak at 440 cm^−1^ for that sample, which was attributed to structural distortion [24]. Although the peak at 1050 cm^−1^ (Figure 1c) has not been defined in the literature, the formation the additional peak for (P)TiO_2_ indicates that a structural change occurred due to the plasma treatment. A similar change in the Raman spectra of (R)TiO_2_, (P-R)TiO_2_, and (R-P)TiO_2_ was not apparent, suggesting that the reduction/passivation may constrain the effect of plasma on the TiO_2_.

**Figure 2:**
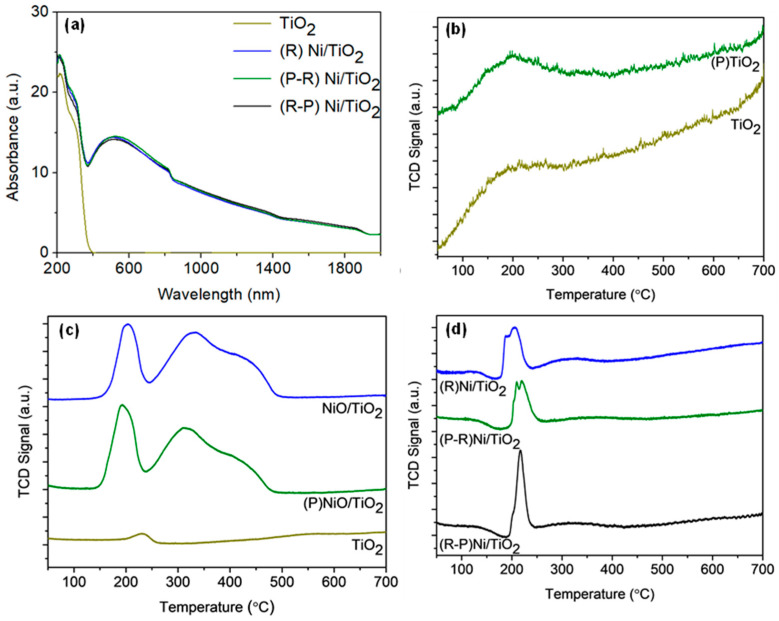
(**a**) UV-vis-NIR absorbance of TiO_2_ and pretreated Ni/TiO_2_; (**b**) CO_2_-TPD of TiO_2_ and plasma-treated TiO_2_ ((P)TiO_2_); H_2_-TPR of (**c**) TiO_2_ and NiO/TiO_2_ and (**d**) pretreated Ni/TiO_2_. R = reduced/passivated, P = plasma treated.

As the photothermal CO_2_ hydrogenation reaction utilises light to heat conversion, the light absorbance of Ni/TiO_2_ was studied by UV-vis-NIR spectroscopy (Figure 2a). The as-prepared TiO_2_ absorbed light in the UV region and exhibited a bandgap of 3.4 eV. The calculated bandgap was consistent with the literature, particularly for anatase TiO_2_ at ~3.2 eV. [33,34], which was the dominant phase present in the materials. Upon Ni addition and pretreatment, the light absorbance extends from the UV region into the visible and near infrared. The absorbance in the Vis and NIR regions can be attributed to the presence of metallic Ni deposits, consistent with group VIII metals [35]. Specifically, the broadband absorbance is driven by intraband transition within overlaid partially filled 3d sub-orbitals [36]. All Ni/TiO_2_ catalysts exhibited full spectrum absorbance, coinciding with the spectrum profile of the Xenon lamp, which allows utilisation of the full solar spectrum for the photothermal CO_2_ reaction.

To understand the impact of plasma treatment on the support, CO_2_-TPD analyses were conducted on the neat TiO_2_ (Figure 2b). A CO_2_ desorption peak was centred at 190 °C and 194 °C for TiO_2_ and (P)TiO_2_, respectively, which indicates medium-strength basic sites [37]. The desorption temperature is favourable, as it is within the temperature range of photothermal CO_2_ methanation. Relative to TiO_2_, the area under the peak for (P)TiO_2_ increased by 11%, indicating a mild increase in CO_2_ adsorption and the effectiveness of plasma treatment in inducing defects in TiO_2_. Defects present on TiO_2_ can behave as sites for CO_2_ adsorption [38]. Subsequently, the increase in CO_2_ desorption peak area can be a contributing factor to the improved activity.

The reducibility of NiO/TiO_2_ and pretreated Ni/TiO_2_ catalysts was characterised by H_2_-TPR (Figure 2c,d). As shown in Figure 2c, the as-prepared TiO_2_ has a low intensity peak between 200–250 °C, which may arise from the partial reduction in TiO_2_ [39] or the removal of residual surface species resulting from the flame synthesis. For NiO/TiO_2_, peaks are observed at ~205 °C, 335 °C, and 425 °C. The ratio of integrated H_2_-TPR peak area was 0.97:1 for (P)NiO/TiO_2_:NiO/TiO_2_, which indicated that slightly less H_2_ was required to reduce (P)NiO/TiO_2_. The lower temperature reduction peaks for NiO/TiO_2_ can be attributed to the reduction in NiO that weakly interacts with TiO_2_. The reduction in nickel oxide occurs at ~400 °C [40]. The broad peak extending up to ~500 °C can be attributed to NiO with a strong NiO–TiO_2_ interaction [41]. After plasma treatment, (P)NiO/TiO_2_ (Figure 2c) displayed a slight decrease in reduction temperature of all peaks to ~195 °C, 310 °C, and 400 °C, implying an ease in reducibility and a slight variation to the metal-support interaction. An increase in the ratio of the lowest temperature peak (~200 °C) to the higher reduction peak (~250 °C to ~500 °C) was observed for (P)NiO/TiO_2_. The peak at ~205 °C for NiO/TiO_2_ accounted for 27% of its reduction profile, while the peak at ~195 °C for (P)NiO/TiO_2_ accounted for 32% of its reduction profile. The 5% increase in the low temperature peak in (P)NiO/TiO_2_ relative to NiO/TiO_2_ depicts an increase in NiO with weak metal-support interaction due to plasma treatment.

Following reduction/passivation, the Ni/TiO_2_ was reducible at temperatures below 250 °C (Figure 2d). The oxygen passivation was conducted to prevent uncontrolled oxidation after catalyst reduction. The decrease in the reduction temperature post-passivation, relative to as-prepared NiO/TiO_2_, is important, as the onset temperature reached in the photothermal system is approximately 200 °C [16]. The post-passivation reduction peaks for (R)Ni/TiO_2_ and (P-R)Ni/TiO_2_ can be deconvoluted into two peaks ((R)Ni/TiO_2_: ~190 °C and 205 °C, (P-R)Ni/TiO_2_: ~210 °C and 220 °C. The post-passivation reduction profile for (R-P)Ni/TiO_2_ was different, with only one reduction peak present at ~215 °C and a shoulder at 200 °C. These peaks are indicative of the re-reduction in the surface oxidised Ni deposits after passivation. The peak ratio, integrated from ~180 °C to ~270 °C and normalised to (R)Ni/TiO_2_, was (R-P)Ni/TiO_2_ (1.01) > (R)Ni/TiO_2_ (1) > (P-R)Ni/TiO_2_ (0.83). The extent of re-oxidation from the oxygen passivation was calculated: (R-P)Ni/TiO_2_ (7.5%) ≈ (R)Ni/TiO_2_ (7.4%) > (P-R)Ni/TiO_2_ (6.3%). Whilst the percent oxidation was comparable between the catalysts, a clear difference in profile features is evident for the (R-P)Ni/TiO_2_ compared to (R)Ni/TiO_2_ and (P-R)Ni/TiO_2_, where two reduction peaks are present, whilst in the case of the (R-P)Ni/TiO_2_ sample, a peak with a lower temperature shoulder is evident. This may be an impact of the re-reduction in different Ni sizes (vide infra). The change in reduction profile for (R-P)Ni/TiO_2_ compared to (R)Ni/TiO_2_ and (P-R)Ni/TiO_2_ indicates that the plasma treatment had the most significant impact on the Ni deposits when implemented after reduction and passivation. However, (P-R)Ni/TiO_2_ required the least H_2_ for re-reduction and subsequently ease of catalyst activation.

**Figure 3:**
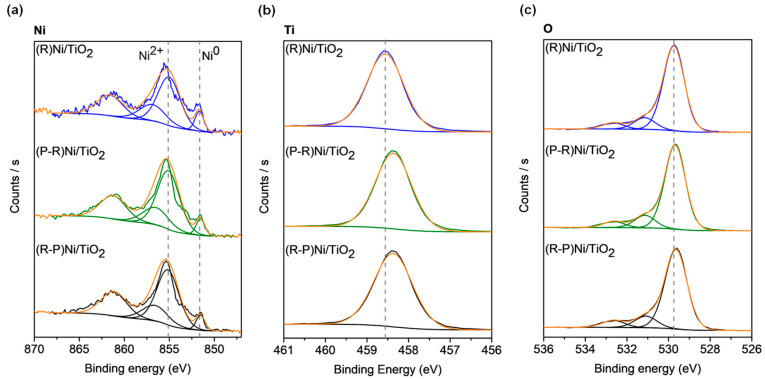
XPS spectra of pretreated Ni/TiO_2_: (**a**) Ni 2p_3/2_, (**b**) Ti 2p, and (**c**) O 1s. R = reduced/passivated, P = plasma treated.

XPS was conducted on the Ni/TiO_2_ to examine their surface oxidation state (Figure 3). Peaks corresponding to Ni^2+^ (nickel oxide) and Ni^0^ (metallic nickel) were identified as seen in Figure 3a, while the unlabelled peaks (at 861.4 eV and 856.6 eV) are satellite peaks [42]. The ratio of Ni^2+^ to Ni^0^ was calculated to be (R-P)Ni/TiO_2_ (6.0) > (R)Ni/TiO_2_ (4.6) > (P-R)Ni/TiO_2_ (4.3). The ratio was consistent with the H_2_-TPR results for Ni/TiO_2_ (Figure 2d) for which (P-R)Ni/TiO_2_ had the lowest quantity of NiO. As is evident in Figure 3b, only a peak corresponding to Ti^4+^ was present in all samples. A shift in the binding energy of Ti^4+^ was observed as a consequence of the impact of plasma, indicating the presence of oxygen vacancy (Ti^3+^) formation [43]. The shift in the Ti^4+^ peak for (P-R)Ni/TiO_2_ (from 458.6 eV to 458.4 eV) and (R-P)Ni/TiO_2_ (from 458.6 eV to 458.4 eV), compared to (R)Ni/TiO_2_ indicates a change in Ti oxidation state arising from plasma treatment. The O 1s spectrum of (R)Ni/TiO_2_ can be deconvoluted to lattice oxygen (529.7 eV), -OH bond (531.2 eV), and adsorbed water (532.7 eV) [44,45]. Corresponding shifts of the oxygen lattice from plasma treatment were observed in (P-R)Ni/TiO_2_ and (R-P)Ni/TiO_2_, from 529.7 eV in (R)Ni/TiO_2_ to 529.6 eV. A slight decrease in oxygen lattice peak position due to plasma treatment has been previously observed [46].

**Figure 4:**
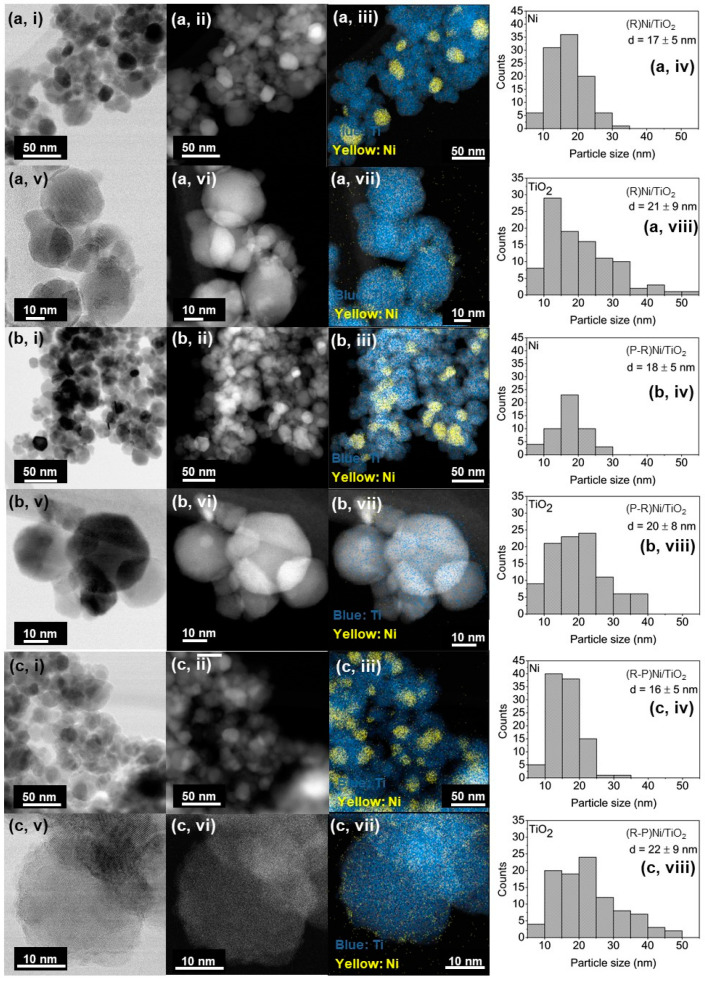
TEM images and EDS mapping of (**a**) (R)Ni/TiO_2_, (**b**) (P-R)Ni/TiO_2_ and (**c**) (R-P)Ni/TiO_2_ including: (**i**,**v**) bright field images; (**ii**,**vi**) dark field images; (**iii**,**vii**) EDS mapping; and (**iv**,**viii**) particle size distribution histograms with range of particle counts = 50–100. R = reduced/passivated, P = plasma treated.

To further understand the interaction between Ni and the TiO_2_ support, TEM images were taken and EDS mapping was performed (Figure 4). The Ni deposits display little difference in size between the (R)Ni/TiO_2_ (17 ± 5 nm) and (P-R)Ni/TiO_2_ (18 ± 5 nm) samples, consistent with the XRD crystallite sizes (Table 1). The (R-P)Ni/TiO_2_ sample also exhibited Ni deposits of a similar size (16 ± 5 nm). However, also present were additional finely dispersed Ni deposits. The sizes of the fine Ni deposits were not able to be accurately measured and, as such, were not included in the associated histogram (Figure 4).

Characterisation of the catalyst properties indicates that plasma treatment modifies the TiO_2_. The modification is evident in changes to the surface texture of the TiO_2_ (Figure 4) and the shift in the Ti 2p peak shift in the XPS spectra (Figure 3) for the plasma-treated samples. Plasma treatment also leads to an increase in CO_2_ adsorption by the neat TiO_2_ (Figure 2b) and structural modification as illustrated by the emergence of a new peak for (P)TiO_2_ in the Raman spectra (Figure 1). Plasma treatment affected the Ni deposit properties, impacting the reducibility of (R-P)Ni/TiO_2_ (Figure 1c,d). To understand the impact of the altered characteristics of the Ni/TiO_2_ catalyst arising from the plasma treatment, the activity/selectivity of the catalysts for CO_2_ methanation under (i) photothermal (batch-circulated reactor system) and (ii) decoupled photo and thermal (continuous flow reactor system) conditions was evaluated.

### 3.2. CO_2_ Methanation Results

**Figure 5:**
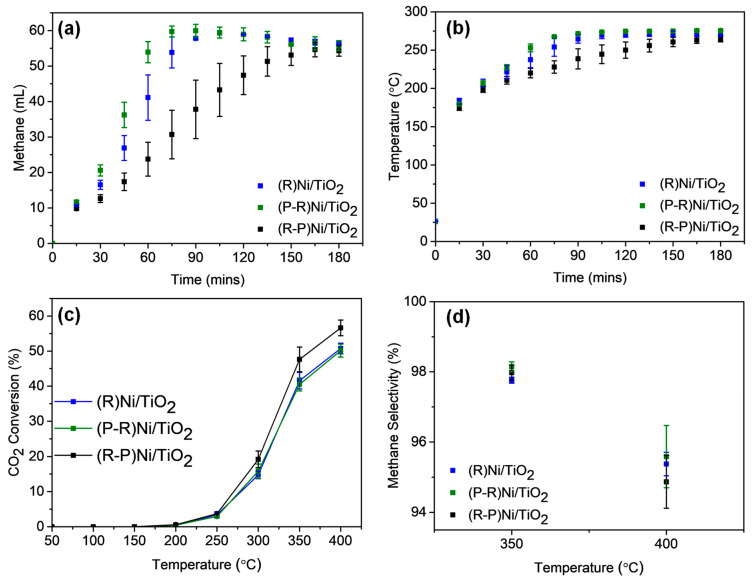
The influence of Ni/TiO_2_ catalyst pretreatment on (**a**) methane formation and (**b**) the temperature profile during photothermal CO_2_ methanation in a batch-circulated reactor system. Catalyst loading = 100 mg; Initial reactant pressure = ~15 kPa CO_2_ and ~60 kPa H_2_; illumination provided by a 300 W Xenon lamp. The influence of Ni/TiO_2_ catalyst pretreatment on (**c**) CO_2_ conversion and (**d**) methane selectivity during decoupled photo-and-thermal CO_2_ methanation in a continuous flow reactor system. Catalyst loading = 100 mg; reacting gas flow rate = 4 mL/min CO_2_ and 16 mL/min H_2_; illumination provided by a 300 W Xenon lamp. Thermal only reaction indicated by the filled markers (●). R = reduced/passivated, P = plasma treated.

The effects of the pretreatment conditions on catalyst activity for the photothermal methanation reaction within the batch-circulated reactor system are shown in Figure 5a,b.

Within the batch-circulated reactor system, the methane formation trend followed the order: (P-R)Ni/TiO_2_ > (R)Ni/TiO_2_ > (R-P)Ni/TiO_2_ (Figure 5a). (P-R)Ni/TiO_2_ and (R)Ni/TiO_2_ required 75 min and 90 min, respectively, to achieve maximum CH_4_ formation with ≈ 100% selectivity (with trace CO). A longer time period was required for methane formation to stabilise in the case of (R-P)Ni/TiO_2_. The temperature profile for each of the Ni/TiO_2_ samples is included in Figure 5b. The temperature for all catalysts reached 175–200 °C within the first 15 min due to the initial photothermal heating of the catalyst combined with the later exothermic heat from methane formation. Given the Ni in the pretreated Ni/TiO_2_ catalysts was reducible below 250 °C (Figure 2d) following reduction and passivation of the catalysts, it is anticipated that catalysts are converted to their reduced form during the batch reaction. Absorbance by the Ni/TiO_2_ catalysts in the infrared region contributed to the localised heating [47]. Consequently, the capacity of the catalysts for full spectrum absorbance was beneficial for the reaction. With increasing methane production, the temperature further increased and reached 250–280 °C after 3 h. The further temperature increase arising from the exothermic nature of methanation can promote better catalyst reduction and contribute to greater methane production [16]. Both (P-R)Ni/TiO_2_ and (R)Ni/TiO_2_ exhibit a similar temperature profile while the temperature profile for (R-P)Ni/TiO_2_ was generally lower. It is apparent that the temperature profile can be correlated to methane formation with (P-R)Ni/TiO_2_ achieving the highest temperature, followed by (R)Ni/TiO_2_ and (R-P)Ni/TiO_2_.

The influences of light and heat were decoupled in the continuous flow reactor system, where a furnace (separate from the light source) was used to heat the catalyst bed. CO_2_ conversion and methane selectivity for thermal-only input are shown in Figure 5c,d. CO_2_ conversion under thermal-only conditions was observable from 200 °C onwards, which is consistent with the findings from the batch photothermal catalytic reactor. A significant increase in CO_2_ conversion was observed with increasing temperature for all catalysts, particularly from 300 °C to 350 °C, with methane as the sole product up to 350 °C. A significant difference in CO_2_ conversion is not apparent over the temperature range 250–400 °C for any of the catalysts. Despite the different pretreatments inducing variations to the catalyst properties, such as reducibility and defect density, the CO_2_ conversion and methane selectivity were not altered. The indifference indicates that the catalyst modification was only effective under photothermal conditions.

Adding illumination to the thermal stimulus can provide better understanding on the influence of light on the reaction, particularly across the UV-visible region. The addition of light, Figure S5, to the thermal conditions did not lead to any significant increase in CO_2_ conversion or change to product selectivity. Subsequently, photothermal CO_2_ conversion by the Ni/TiO_2_ catalysts appears to be thermally dominant. Under both conditions—thermal-only and light-plus-thermal conditions—methane selectivity decreased from 350 °C onwards in favour of CO formation due to the reverse-water gas shift reaction [11].

The thermal dominance during photothermal CO_2_ conversion indicates that the absorbance of light from the NIR portion of the spectrum is crucial for reaction feasibility. While catalyst modifications induced by pretreatment, such as greater CO_2_ adsorption, TiO_2_ defects, and a change in metal-support interaction, did not promote greater CO_2_ conversion under the decoupled photo-and-thermal condition (continuous flow reactor), they do significantly influence the photothermal catalytic activity (cyclic batch reactor). Importantly, the order in which the plasma and reduction/passivation treatments were applied influenced the catalytic activity of the Ni/TiO_2_.

Others have examined defects generated in TiO_2_ as a consequence of plasma treatment. Bharti et al. reported that plasma treatment in an air environment could induce Ti^3+^ and oxygen vacancies in a TiO_2_ thin film [46]. The higher activity of (P-R)Ni/TiO_2_ compared to (R-P)Ni/TiO_2_ in our work can be related to additional defects formed and their ensuing stability arising from the different pretreatment sequences. Chen et al. found that plasma treating Pt/CeO_2_ followed by 20% H_2_/N_2_ thermal treatment led to higher toluene catalytic oxidation, compared to thermal then plasma treatment. The plasma treatment affected the surface oxygen defects and Pt-Ce interaction. When thermally treating the catalyst after plasma treatment, more defects could be generated via greater hydrogen dissociation on the plasma-generated defect sites [48]. In our case, while the impact of plasma treatment was most prominent for (R-P)Ni/TiO_2_, the changes were not necessarily beneficial for enhancing photothermal CO_2_ hydrogenation relative to (R)Ni/TiO_2_. The thermal treatment can stabilise the catalyst structure and subsequently minimise the impact of any ensuing plasma treatment [24]. Consequently, the results suggest that the most effectual approach for the Ni/TiO_2_ catalyst is to thermally treat the defected catalyst to stabilise the defects generated from the plasma treatment.

## 4. Conclusions

The influence of Ni/TiO_2_ catalyst pretreatment strategy on the generation and stabilisation of surface defects active for the photothermal CO_2_ methanation reaction was examined. The sequence of two pretreatment steps, involving a He-plasma stage and a reduction/passivation stage, were switched, with the ensuing impact on defect generation and, in turn, catalyst activity, assessed. The catalyst comprised a TiO_2_ support prepared via FSP which was loaded with a Ni catalyst. When the as-prepared NiO/TiO_2_ was treated with plasma, the subsequent reduction/passivation step stabilised the plasma-generated defects on the TiO_2_ support, as evident from XPS, and Raman analysis. This increase in defects resulted in an increased CO_2_ interaction (shown in the CO_2_-TPD of the plasma-treated TiO_2_). Ultimately, these increased surface TiO_2_ defects resulted in higher photothermal catalytic performance. When reduction/passivation was performed prior to plasma treatment, the reduction/passivation imbued the NiO/TiO_2_ with a greater resistance to subsequent defecting by the plasma treatment. It was evident, through H_2_-TPR of the Ni/TiO_2_ samples (after reduction and passivation), that a change in metal–support interaction occurred as a result of plasma treatment after reduction. Deconvolution of the light and heat effects demonstrated that the photothermal catalytic reaction was thermally dominant with the light as a heat source. The work highlights the importance of the material pretreatment strategy when using plasma as a means to generate active defect sites within a catalyst. If not performed correctly, the potential benefits of plasma treatment can be lost.

## Figures and Tables

**Table 1:** BET specific surface area and crystallite size (TiO_2_, NiO, and Ni) of as-prepared and pretreated Ni/TiO_2_.

Catalyst	Pretreatment ^a^	S_BET_ (m^2^/g) ^b^	TiO_2_ Crystal Size (nm) ^c^	NiO Crystal Size (nm) ^c^	Ni Crystal Size (nm) ^c^
TiO_2_	N/A	104	22.0	N/A	N/A
NiO/TiO_2_	N/A	66	21.0	7.7	N/A
(R)Ni/TiO_2_	Reduced	N.D.	23.6	N/A	13.1
(P)NiO/TiO_2_	Plasma treated	75	21.0	9.3	N/A
(P-R)Ni/TiO_2_	Plasma treated then reduced	N.D.	22.6	N/A	13.7
(R-P)Ni/TiO_2_	Reduced then plasma treated	N.D.	23.0	N/A	12.7

^a^ “Reduced” refers to “reduction/passivation”. ^b^ BET specific surface area. ^c^ Calculated from 100% intensity peak of XRD pattern (Figure S2).

## Data Availability

The data presented in this study are available in this article and the Supplementary Information.

## Supplementary Materials

The following are available online at https://www.mdpi.com/article/10.3390/ma14154195/s1, Figure S1: N_2_ adsorption/desorption isotherm and pore size distribution of (a,d) TiO_2_, (b,e) NiO/TiO_2_, and (c,f) (P)NiO/TiO_2_, respectively, Figure S2: XRD patterns of (a) TiO_2_ and NiO/TiO_2_ and (b) Ni/TiO_2_ catalysts following different pretreatment approaches. R = reduced/passivated, P = plasma treated. JCPDS: 00-044-1159 (NiO), 00-004-0850 (Ni), 00-021-1272 (anatase TiO_2_), and 98-005-3997 (rutile TiO_2_), Figure S3: (a) EPR and (b) Raman spectra of as-prepared TiO_2_ following different pretreatment approaches. R = reduced/passivated, P = plasma treated, Figure S4: The influence of Ni/TiO_2_ catalyst pretreatment on (a) CO_2_ conversion and (b) methane selectivity during decoupled photo and-thermal CO_2_ methanation in a continuous flow reactor system. Catalyst loading = 100 mg; Reacting gas flow rate = 4 mL/min CO_2_ and 16 mL/min H_2_; illumination provided by a 300 W Xenon lamp. Thermal only reaction indicated by the filled markers (●). Photo-and-thermal reaction indicated by the open markers (○). R = reduced/passivated, P = plasma treated.
