# Supplementary material for: Plasma-Induced Catalyst Support Defects for the Photothermal Methanation of Carbon Dioxide
Source: Materials (Basel). 2021 Jul 28;14(15):4195. doi: 10.3390/ma14154195 (PMC8347005; doi:10.3390/ma14154195)
Supplement: Supplementary file 1 [file materials-14-04195-s001.zip › materials-1268515-supplementary.pdf]

materials

MDPI

Supplementary Material

Plasma-Induced Catalyst Support Defects for the Photothermal Methanation of Carbon Dioxide

Salina Jantarang, Simone Ligori, Jonathan Horlyck, Emma C. Lovell *, Tze Hao Tan, Bingqiao Xie, Rose Amal and Jason Scott *

Particles and Catalysis Research Group, School of Chemical Engineering, The University of New South Wales, Sydney, NSW 2052, Australia; salina.j@chula.ac.th (S.J.); simligori@gmail.com (S.L.); j.horlyck@outlook.com (J.H.); tze_hao.tan@unsw.edu.au (T.H.T.); bingqiao.xie@unsw.edu.au (B.X.); r.amal@unsw.edu.au (R.A.)

* Correspondence: e.lovell@unsw.edu.au (E.C.L.); jason.scott@unsw.edu.au (J.S.)

Citation: Jantarang, S.; Ligori, S.; Horlyck, J.; Lovell, E.C.; Tan, T.H.; Xie, B.; Amal, R.; Scott, J. Plasma-Induced Catalyst Support Defects for the Photothermal Methanation of Carbon Dioxide. *Materials* **2021**, *14*, 4195. https://doi.org/10.3390/ma14154195

Academic Editor: Kai Yan

Received: 4 June 2021
Accepted: 20 July 2021
Published: 28 July 2021

Publisher's Note: MDPI stays neutral with regard to jurisdictional claims in published maps and institutional affiliations.

Copyright: © 2021 by the authors. Licensee MDPI, Basel, Switzerland. This article is an open access article distributed under the terms and conditions of the Creative Commons Attribution (CC BY) license (http://creativecommons.org/licenses/by/4.0/).

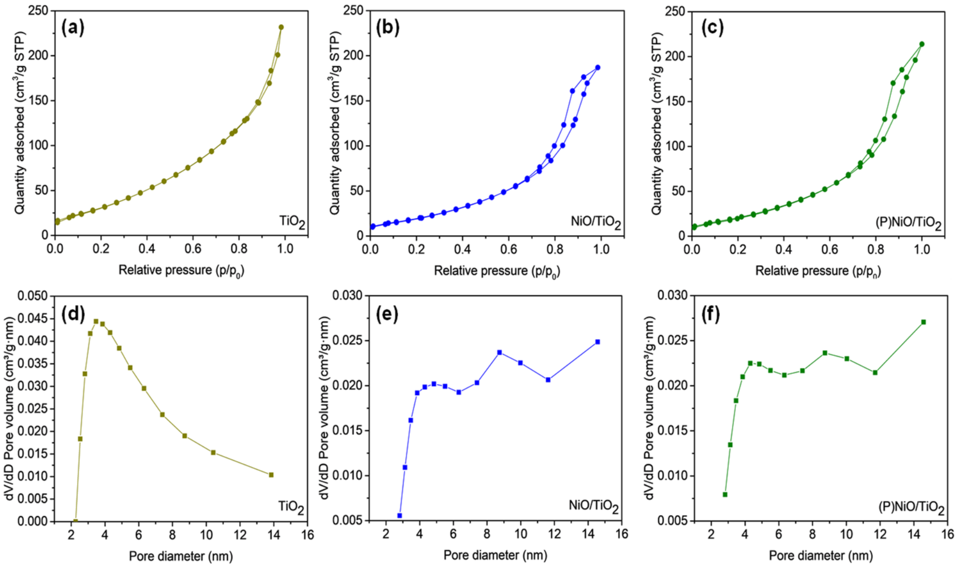

Figure S1. N_2 adsorption/desorption isotherm and pore size distribution of (**a**,**d**) TiO_2, (**b**,**e**) NiO/TiO_2, and (**c**,**f**) (P)NiO/TiO_2, respectively.

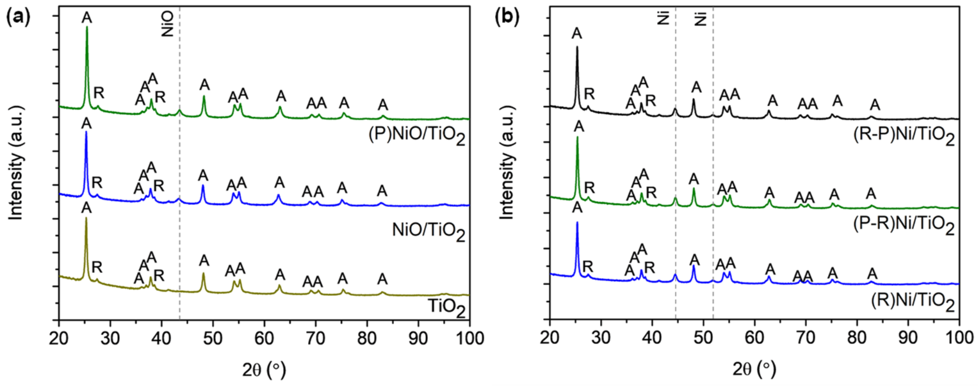

Figure S2. XRD patterns of (**a**) TiO_2 and NiO/TiO_2 and (**b**) Ni/TiO_2 catalysts following different pretreatment approaches. R = reduced/passivated, P = plasma treated. JCPDS: 00-044-1159 (NiO), 00-004-0850 (Ni), 00-021-1272 (anatase TiO_2), and 98-005-3997 (rutile TiO_2).

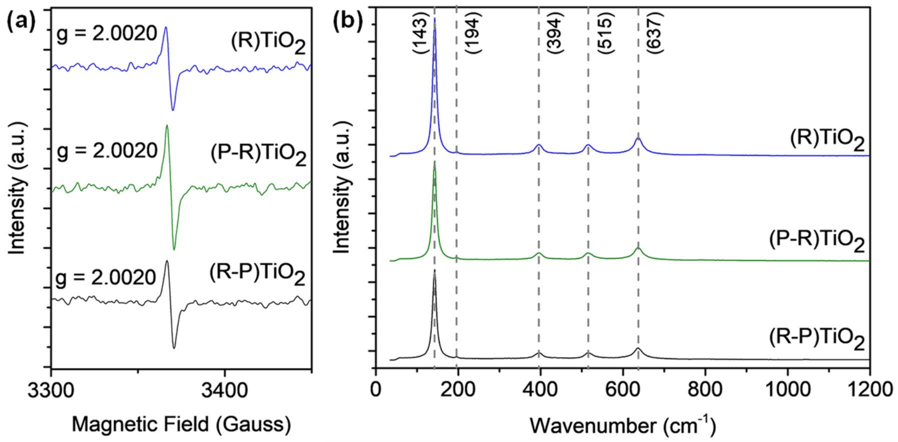

Figure S3. (**a**) EPR and (**b**) Raman spectra of as-prepared TiO_2 following different pretreatment approaches. R = reduced/passivated, P = plasma treated.

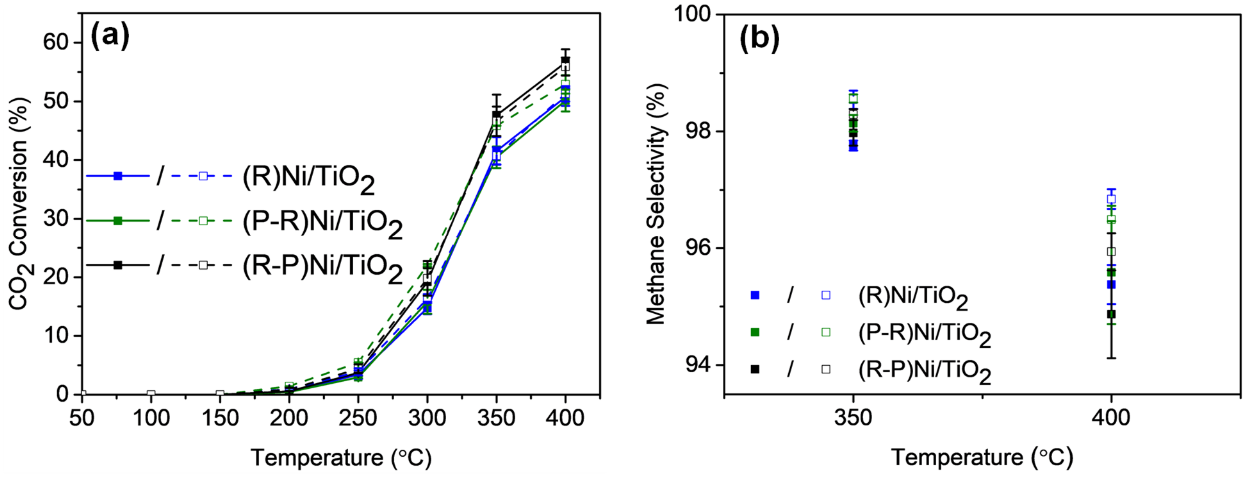

Figure S4. The influence of Ni/TiO_2 catalyst pretreatment on (**a**) CO_2 conversion and (**b**) methane selectivity during decoupled photo-and-thermal CO_2 methanation in a continuous flow reactor system. Catalyst loading = 100 mg; Reacting gas flow rate = 4 mL/min CO_2 and 16 mL/min H_2; illumination provided by a 300 W Xenon lamp. Thermal only reaction indicated by the filled markers (●). Photo-and-thermal reaction indicated by the open markers (○). R = reduced/passivated, P = plasma treated.
